# Chronic Inflammation in Immune Aging: Role of Pattern Recognition Receptor Crosstalk with the Telomere Complex?

**DOI:** 10.3389/fimmu.2017.01078

**Published:** 2017-09-04

**Authors:** Shyam Sushama Jose, Kamila Bendickova, Tomas Kepak, Zdenka Krenova, Jan Fric

**Affiliations:** ^1^Cellular and Molecular Immunoregulation Group (CMI), Center for Translational Medicine (CTM), International Clinical Research Center (ICRC), St. Anne’s University Hospital Brno, Brno, Czechia; ^2^Department of Biology, Faculty of Medicine, Masaryk University, Czechia; ^3^Pediatric Oncology Translational Research (POTR), International Clinical Research Center (ICRC), St. Anne’s University Hospital Brno, Brno, Czechia; ^4^Pediatric Hematology and Oncology, University Hospital Brno, Brno, Czechia

**Keywords:** pattern recognition receptor signaling, telomere shortening, inflammaging, myelopoiesis, NF-κB, toll-like receptor signaling

## Abstract

Age-related decline in immunity is characterized by stem cell exhaustion, telomere shortening, and disruption of cell-to-cell communication, leading to increased patient risk of disease. Recent data have demonstrated that chronic inflammation exerts a strong influence on immune aging and is closely correlated with telomere length in a range of major pathologies. The current review discusses the impact of inflammation on immune aging, the likely molecular mediators of this process, and the various disease states that have been linked with immunosenescence. Emerging findings implicate NF-κB, the major driver of inflammatory signaling, in several processes that regulate telomere maintenance and/or telomerase activity. While prolonged triggering of pattern recognition receptors is now known to promote immunosenescence, it remains unclear how this process is linked with the telomere complex or telomerase activity. Indeed, enzymatic control of telomere length has been studied for many decades, but alternative roles of telomerase and potential influences on inflammatory responses are only now beginning to emerge. Crosstalk between these pathways may prove to be a key molecular mechanism of immunosenescence. Understanding how components of immune aging interact and modify host protection against pathogens and tumors will be essential for the design of new vaccines and therapies for a wide range of clinical scenarios.

## Introduction

Aging is a complex process that involves a gradual decline in critical cellular processes, signaling pathways, and regulatory mechanisms, leading to eventual disruption of tissue homeostasis (1). Accumulation of cell functional defects over time, commonly termed “senescence,” is a driving force of human aging and confers increased risk of cardiovascular and neurodegenerative disorders, as well as autoimmune disease and infection (2). Cellular senescence-associated changes affect numerous processes including proliferation or changes in secretome. Recent studies have shown that chronic inflammation contributes to pathological aging by promoting stem cell exhaustion, impairment of cellular communication, and somatic cell loss of the repetitive nucleotide sequences known as telomeres that form protective “caps” at the ends of chromosomes (1).

To maintain telomere length and protect chromosomes against damage, cell types with high proliferative capacity such as hematopoietic progenitors (3, 4) and effector leukocytes (5, 6) employ the inducible enzyme telomerase to maintain telomere length. In addition, the multiprotein complex shelterin coordinates the formation of protective “loop” structures that prevent telomere ends from being recognized as DNA breaks (7). While a large number of studies have investigated telomere length and telomerase activity as prognostic biomarkers in human cancer, this review instead focuses on the potential interactions between inflammation and telomere biology in immunological aging. Indeed, telomerase activity is now known to be strongly influenced by leukocyte proliferative activity, ongoing inflammation, and production of reactive oxygen species (ROS), but the molecular basis of these effects is not yet fully understood. In particular, the transcription factor NF-κB, which has long been associated with pattern recognition receptor (PRR) signaling and inflammation, has recently been identified as an important regulator of the telomere complex. Better definition of potential immune crosstalk with telomerase activity may therefore yield a range of novel therapeutic targets for intervening in age-related and inflammatory pathologies.

## Immunosenescence

Effective host immunity is essential for the maintenance of tissue homeostasis and health, but both innate and adaptive responses are subject to natural age-related functional decline termed “immunosenescence” (8). Key features of immunosenescence include a progressive loss of naïve T cells and accumulation of memory T cells in body tissues (9–11) as well as gradual deterioration of innate leukocyte defense mechanisms (8, 12). In this review, we focus mainly on senescence-associated changes in the innate immune compartment, which mediates first line of defense against infections. Senescence impacts on several major mechanisms of innate protection against pathogens, including phagocytosis and ROS production by neutrophils, as well as toll-like receptor (TLR) expression and cytokine release by macrophages and dendritic cells. Key defects in innate cell activity associated with senescence have been reviewed elsewhere (8, 12, 13). These include a range of deficits in myeloid cell functions, which are governed primarily *via* PRR signaling and have been identified as displaying significant impairment in various senescence-related disorders.

Myeloid cell-derived biomarkers of immunosenescence reportedly include increased production of the cytokines interleukin 6 (IL-6) and tumor necrosis factor α (TNF-α), which correlate with elevated serum levels of C-reactive protein to predict increased patient frailty and higher overall rates of mortality (14). IL-6 and TNF-α are produced mainly by tissue macrophages and T cells and have already been implicated in multiple age-related disorders including osteoarthritis, cardiovascular disease, autoimmunity, and neurodegeneration (15). Both IL-6 and TNF-α are able to increase telomerase activity through NF-κB, STAT1, and STAT2 activation (16). However, the mechanism by which these mediators of inflammation impact on the aging process remains poorly defined. For example, serum IL-6 levels have previously been identified as a predictive biomarker of mortality risk in the elderly (17, 18), but this cytokine has also been shown to exert anti-inflammatory effects in certain age-related pathologies including rheumatoid arthritis (19). Therefore, additional studies will be required to identify the molecular mediators involved so that these can be targeted by future therapeutic strategies.

Although immunosenescence occurs naturally as the human body ages, early activation of senescence pathways has been observed in a wide range of human disorders (20, 21). Immunosenescence is also associated with hematopoietic dysfunction, leading to a decline in leukocyte numbers and function across both the innate and adaptive arms of the immune system (22–24). These detrimental effects are typically associated with prolonged, low-grade infection or inflammation (25, 26) and/or persistent infection by pathogens including cytomegalovirus (27, 28). Previous studies have indicated that low-grade inflammation induced by genetic deletion of NF-κB subunit can confer telomere dysfunction (29) and that bone marrow-derived macrophages from aged mice exhibit short telomeres and impaired inflammatory signaling (30). It seems likely therefore that mechanisms of telomere maintenance impact on immune function and *vice versa*, in particular, *via* interactions with the enzyme telomerase. Indeed, emerging data indicate that telomerase likely exerts a range of additional functions that could significantly impact on hematopoiesis and mitochondrial ROS production during age-related immune decline.

## “Inflammaging”

Immunosenescence is strongly driven by persistent infections and/or tissue inflammation (1, 31), leading some investigators to term this process “inflammaging” to better distinguish pathological events from natural age-related decline (20, 21, 32). In some settings, inflammaging is a consequence of unresolved “sterile” inflammation resulting from organelle/molecule damage, inappropriate immune signaling, and autoantigen (33). Although inflammation is primarily maintained by secreted cytokines, as already reviewed elsewhere (34, 35), another important factor is damaged cell/tissue release of stimulatory molecules that can activate myeloid cells by signaling through PRRs such as TLRs. PRRs recognize specific pathogen-associated molecular patterns (PAMPs) as well as host-derived damage-associated molecular patterns (DAMPs) that are produced by stressed, malfunctioning, and injured cells. Several DAMPs released by damaged mitochondria (36, 37) and nuclei (38, 39) or derived from the cytoplasm (40, 41) have already been linked with inflammaging. Failure to resolve low-grade inflammation can result in both innate and adaptive immune responses to self-antigens, progressive tissue damage, and pathological cellular aging. Accordingly, sustained activation of PRR pathways has already been identified in a number of chronic inflammatory disorders associated with aging (Table 1), and changes in PRR expression and signaling are now widely recognized as critical components of immunosenescence (12, 24). Inflammation-induced immune aging in host tissues is therefore a consequence of multiple detrimental pathways acting in concert over a prolonged period of time.

**Table 1:** Chronic inflammatory diseases with reported telomere shortening, changes in telomerase activity, and a role for PRRs.

Disease category	Pathology/disease type	PRRs associated with disease and cell types affected	Cell-specific telomere shortening	Telomerase activity
Cardiovascular diseases	Atherosclerosis	TLRs (42–45); Mo, MF, DC, MC, aortic tissue	Leukocytes (46)	MF, aortic tissue, ↗ (47, 48)
	Chronic heart failure	TLRs, NLRs (49); MF, heart tissue	Leukocytes (50)	ND

Pulmonary diseases	Chronic obstructive pulmonary disease	TLRs (51); Mo, MF, lung tissue	Leukocytes (52, 53)	ND
	Sarcoidosis	TLR2 (54);BAL	Leukocytes (55, 56)	ND

Hepatic diseases	Non-pathogenic hepatitis	TLRs (57–60); hepatocytes, biliary epithelia, sinusoidal endothelia, MF, Mo	Liver tissue (61)	ND
	Primitive biliary cirrhosis	TLRs (62), Mo	Bile duct (63)	ND

Gastrointestinal diseases	Ulcerative colitis	TLR4, TLR5 (64, 65); mucosa	Leukocytes, mucosa (66–69)	Mucosa, ↗ (70)
	Celiac disease	TLR2, TLR4 (71)	Leukocytes (72)	ND

Joint and muscle diseases	Idiopathic inflammatory myopathies	TLRs, NLRs (73), skeletal muscle, MF, DC	No significant shortening (74)	Skeletal muscle, ↗ (74)
	Rheumatoid arthritis	TLRs (75, 76), synovial tissue	Leukocytes, T cells (77, 78)	Synovial ts., ↗ (79, 80)
	Juvenile idiopathic arthritis	TLRs (81), Mo	Naïve T cells (82, 83)	ND
	Systemic sclerosis	TLRs (84–86), synovial tissue	No significant shortening (87)	PBMCs, ↘ (88)

Other autoimmune conditions	Systemic lupus erythematosus	TLR7, TLR9 (89–91), mesangial cells	Leukocytes (92)	PBMCs, T cells, ↗ (88, 93)

Infectious diseases (chronic infections)	*Helicobacter pylori*	TLR2, TLR4 (94–96), gastric mucosa, gastric epithelial cells	Gastric mucosa (97)	Gastric mucosa, ↗ (98)
	Hepatitis B	TLRs (99, 100), PBMC	Hepatocytes (101)	PBMCs, ↘ (102)

Alcohol, smoking, and obesity-related diseases	Alcohol consumption	TLR4, TLR2 (103, 104), Kupffer cells, lung epithelia	Eosophageal epithelium (105)	ND
	Smoking	TLR4 (103, 106), Lung epithelia	Leukocytes (107, 108)	↗ (109), lung epithelia
	Obesity	TLRs (110, 111), adipose tissue	Leukocytes (108)	ND

*Chronic inflammation plays a major role in progression of various disorders and autoimmune pathologies. This table lists diseases in which shortening of telomeres, changes in telomerase activity, and a role for TLR signaling have been reported. Although direct interaction between these processes has yet to be formally demonstrated, these events have been closely correlated in a range of different disorders and putative mechanisms are now beginning to emerge. While short telomeres have frequently been associated with human disease, telomere length is not always correlated with disease severity*.

*Mo, monocyte; MF, macrophage; DC, dendritic cell; MC, mast cell; BAL, bronchoalveolar lavage; PBMCs, peripheral blood mononuclear cells; PRR, pattern recognition receptor*.

## Hematopoietic Stem Cell Exhaustion in Chronic Inflammation

Natural age-associated changes in innate immune function have already been described in adults older than 40 years (112), whereas early-onset immunosenescence has been associated with various pathologies. Changes in TLR expression and function likely represent key components of both healthy and pathological immune aging (113). In particular, various types of hematopoietic progenitors have been shown to express TLRs (114, 115), which may play direct roles in senescence of the progenitor pool (113, 115). Steady-state differentiation of hematopoietic stem and progenitor cells (HSPCs) into myeloid lineage cells is controlled by growth factors including G-CSF, M-CSF, GM-CSF, and Flt3-L, but can be modified by pro-inflammatory cytokines such as IFN-γ during an immune response (116, 117). Chronic inflammation can also generate massive quantities of DAMPs including calgranulins (S100A8/9), high mobility group box-1 (HMGB1), and serum amyloid A, which can engage PRRs expressed by multiple cell types. Direct TLR stimulation of HSPCs in the bone marrow and circulation may therefore accelerate the immune aging process (113, 118).

Direct roles for HSPCs in inflammation have only recently been described by Griseri et al. who identified progenitor cell infiltration of the gut mucosa in experimental colitis (119). Most studies of PRR function in HSPCs have focused on the small number of cells that circulate in peripheral blood, where these progenitors can detect PRR ligands and enhance extramedullary hematopoiesis during inflammation (118, 119). HSPC stimulation with TLR ligands can potently modulate differentiation pathways and typically favors myeloid cell development (114, 115, 120), but prolonged TLR triggering eventually leads to progenitor exhaustion and loss of self-renewal capacity (121–123). Bone marrow HSPCs can also mediate “emergency hematopoiesis” in response to PRR ligation of DAMPs and PAMPs (114, 124), particularly in the context of bacterial infection (125, 126) or fungal invasion (127, 128). However, inflammatory modulation of hematopoietic activity is not restricted to the blood and bone marrow, since somatic cells and tissues also appear to influence this process (129, 130). It is also important to note that PRR signaling in HSPCs can play a role in cell reconstitution even under resting conditions, since TLR4/TRIF reportedly mediates the steady-state renewal of granulocytes (131). Taken together, these data indicate that inflammation can induce PRR signaling in HSPCs and accelerate/modify cellular differentiation to promote progenitor exhaustion and immune system dysfunction, both of which are important hallmarks of immunosenescence. To what extent stem cell telomeres and telomerase are involved in these events remains unclear, although HSPC skewing toward generation of myeloid-lineage cells has previously been linked with telomere dysfunction (132), and experimental mice lacking the telomerase subunits telomerase reverse transcriptase (TERT) or telomerase RNA component (TERC) exhibit increased myeloid progenitor cell numbers in bone marrow (133).

Formal demonstration of a direct influence of PRRs/inflammation on telomere length/telomerase activity in host leukocytes and stem cells is currently lacking, but experimental data consistent with this concept are continuing to accumulate. Indeed, age-related DNA damage and shortened telomeres have been observed in murine HSCs (134), and senescent progenitor cells with shortened telomeres exhibit increased activity of the pro-inflammatory transcription factor NF-κB (135). TERC-deficient mice also exhibit chromosome instability that enhances signaling through TLR4/NF-κB, leading to increased macrophage expression of pro-inflammatory cytokines and high susceptibility to endotoxin shock (136). These and other influences of PRR signaling on accumulation of DNA damage in host cells have been expertly reviewed elsewhere (137). It seems likely therefore that direct crosstalk between PRRs and telomerase activity will also prove critical to the immunosenescence process in humans. This could have major implications for the design of therapies to maintain effective host immunity in elderly patients and treat various inflammatory disorders. Indeed, immune aging has already been identified as a major determinant of bone marrow progenitor quality and functionality during transplantation (138). Inflammatory DAMP generation and PRR triggering of HSPCs have also been reported to increase pathology in disorders including atherosclerosis (42, 43, 139), colitis (119), and chronic dermatitis (140). Further detrimental effects of inflammation on HSPCs have been observed in models of chronic PRR triggering (117, 126, 141) as well as in human sepsis (142), while age-related change in hematopoietic function have also been shown to confer increased risk of anemic and malignant disorders (143). PRR-driven signaling has now been observed to correlate with altered telomere length or telomerase activity in numerous cell types and tissues from patients with chronic inflammatory disorders (Table 1), but the mechanistic basis of this link has not yet been defined. Despite their disparate origins and diverse pathological features, these diseases share common features of oxidative stress and inflammation together with telomere shortening, suggesting tight associations between inflammatory disorders and cellular senescence across a range of clinical settings.

## Mitochondrial Damage in Inflammaging

Mitochondrial ROS production is a key antimicrobial function of specialized immune cells including macrophages, dendritic cells, and neutrophils. Accordingly, age-related impairment of mitochondrial function can significantly impair host immune responses (144). Increasing age is typically accompanied by decreased mitochondrial output of antimicrobial ROS together with a parallel increase in oxidative stress. While a role for mitochondrial dysfunction in immunosenescence is now well established, the basis of this association may be more complex than initially thought. Recent reports have indicated that DNA release from damaged mitochondria is a major driver of ROS production and inflammation (145, 146) and may therefore promote host immunosenescence *via* a range of different mechanisms (147). ROS accumulation also promotes further mitochondrial dysfunction, oxidative stress, and release of DNA into the cytosol where this can activate the NLRP3 inflammasome (146). While neutrophils exhibit only a short half-life in blood and typically lack TERT expression or telomerase activity (148), during inflammation these cells are a major source of ROS and can reportedly acquire telomerase activity on infiltration of unstable coronary plaques (149). Further studies will now be required to resolve the exact role of cytoplasmic TERT expression in neutrophils that lack TERC (150) and to determine the contribution of these cells to immunosenescent pathology.

Reactive oxygen species have also been strongly implicated in pathological changes in blood vessel structure and function that characterize age-related vascular diseases such as atherosclerosis (151). In this context, Jurk et al. used a genetic model of chronic low-grade inflammation to demonstrate that ROS exacerbate telomere dysfunction (29). It now seems that oxidative stress, mitochondrial damage, and cellular aging are intimately linked in multiple species including yeast (152) and trypanosomes (153), although additional data from animal models and validation in human studies will be required to fully understand this.

## Inflammaging, Telomerase Activity, and Telomere Length

**Figure 1:**
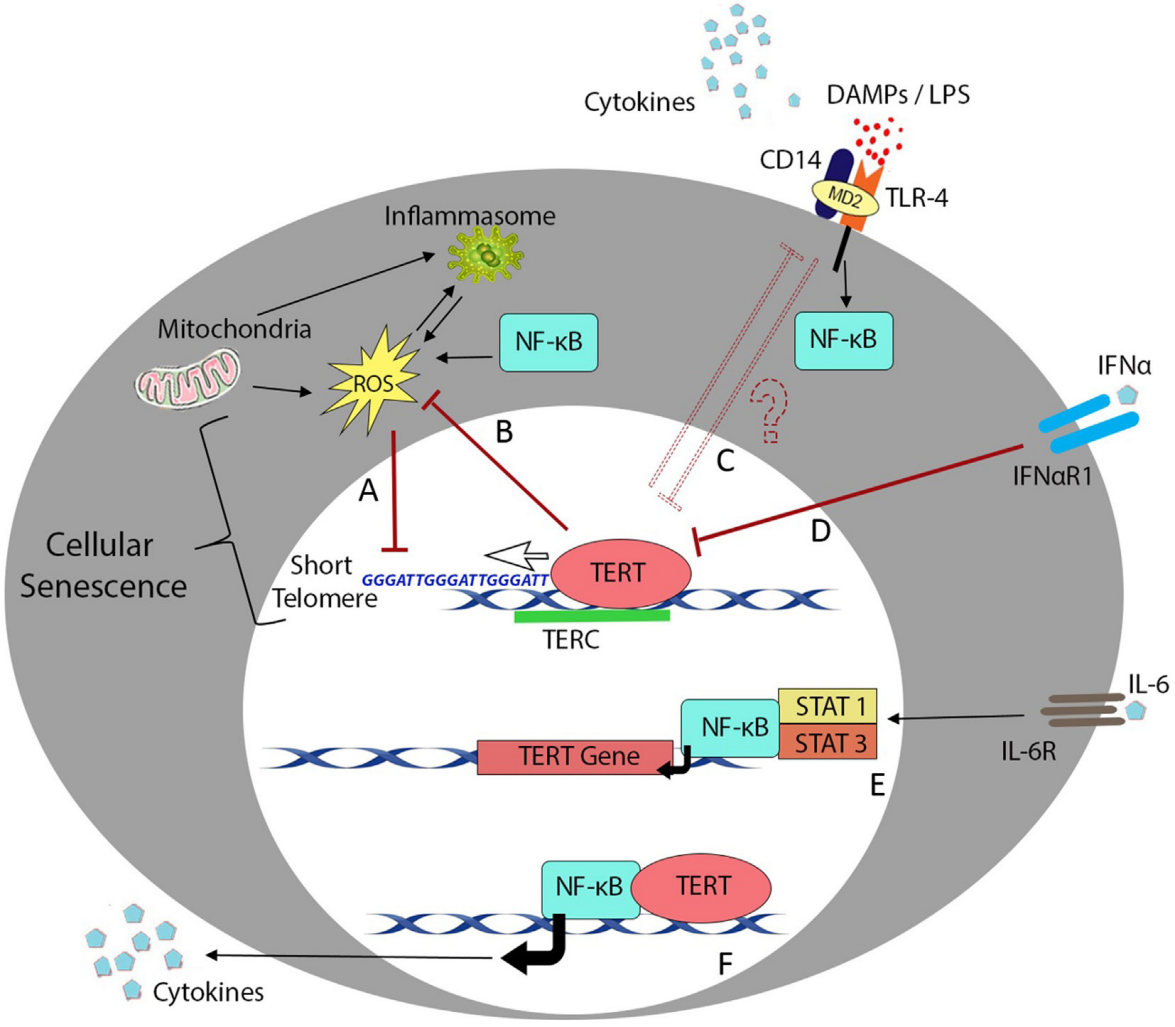
Telomere length and telomerase activity during inflammation. Overview of the major cellular processes linking the telomere complex with inflammatory signaling and immunosenescence. Transcription factor NF-κB plays a crucial role in most inflammatory processes but also interacts with telomere control machinery and putative non-telomeric functions of the telomerase enzyme. **(A)** Low-grade inflammation in nfkb1^−/−^ mice causes increased ROS production and results in telomere dysfunction in mouse hepatocytes and intestinal crypt stem cells (29). **(B)** One of the reported non-telomeric functions of human telomerase enzyme (TERT) is the ability to inhibit endogenous ROS production and regulate oxidative stress in cancer cell lines (155). **(C)** Mice lacking telomerase RNA component (TERC) succumb to LPS administration due to endotoxin shock arising from chromosome instability in splenocytes and macrophages (136). **(D)** Signaling downstream of inflammatory cytokines such as IFN-α plays an important role in downregulation of TERT activity in hematopoietic cells (159). **(E)** In contrast, interleukin (IL)-6 and tumor necrosis factor (TNF)-α reportedly upregulate TERT transcription and telomerase activity through activation and binding of NF-κB in macrophages (47) or NF-κB, STAT1, and STAT3 interactions with the TERT promoter in splenocytes and cancer cells (16, 158). **(F)** Ghosh et al. have also described the ability of TERT to directly regulate NF-κB-dependent gene expression in primary bone marrow blasts from leukemic patients (160).

Telomere shortening during cell division is a critical process in progression to senescence (154), and telomerase may play an important role in immunological aging. Overexpression of telomerase subunit TERT can decrease oxidative stress in cancer cell lines (155), whereas TERT-deficient HSCs are characterized by ROS impairment and functional defects (156). Similarly, chromosome instability arising from TERC deficiency promotes TLR4 stimulation (136), while telomeric repeats (TTAGGG) can inhibit CpG binding to TLR9 to impair innate immune activation (157). Telomerase activity also appears to be subject to modulation by the activity of NF-κB (29) and/or exposure to pro-inflammatory cytokines (16, 158, 159) as summarized in Figure 1. However, it is important to note that telomerase expression level and enzymatic activity do not always directly correlate with senescent status or even telomere length; hence, further studies will be needed to better understand these complex interactions in human cells and tissues.

Even in the absence of NF-κB signaling, prolonged low-grade inflammation is sufficient to induce telomere dysfunction, likely involving accumulation of mitochondrial ROS (29). TERT can integrate numerous upstream signals including Wnt/β-catenin developmental cues (161) and can regulate inflammatory signaling through binding to NF-κB promoters and subsequent transcription of NF-κB-regulated genes including IL-6 and TNF-α (160). This crosstalk is exemplified by an alcoholic liver disease model in which NF-κB was observed to regulate protein expression levels of the catalytic subunit TERT (158), which in turn modulated NF-κB signaling to promote macrophage polarization toward an inflammatory M1 phenotype with increased expression of IL-6 and TNF-α (162). Increased peripheral blood expression levels of IL-6 and TNF-α in patients with metabolic disorders have also been shown to correlate with elevated levels of telomerase activity (163).

The central role of NF-κB in regulating chronic, low-grade inflammation has long been established, but only recently have experimental data begun to indicate a possible role for NF-κB in control of telomerase expression or activity in the context of senescence-associated disorders. For example, Gizard et al. showed that inflammation-induced NF-κB activation regulates TERT expression in macrophages and that human atherosclerotic lesions are characterized by high expression of TERT (47). Disease-associated changes in PRR signaling and telomere biology have also been identified within individual cells or host tissues, including the inflamed gut mucosa in ulcerative colitis (64–70), synovial tissues in rheumatoid arthritis (75, 76, 79, 80), and smoke-exposed lung epithelia (103, 106, 109). However, these features have often been described across multiple separate reports; hence, definitive proof of functional links between these processes is still lacking. Indeed, while short telomeres in leukocytes have been identified as a key component of pathological immune aging (5, 6, 164), direct associations with human senescence have not yet been confirmed, and the majority of relevant mechanistic data have been generated exclusively in mouse models. This is a particular challenge given that mouse telomeres can be up to 10 times longer than their equivalent human sequences despite a much shorter animal lifespan (165). Nonetheless, substantial data have now been obtained using genetically engineered TERC/TERT-knockout mice, which replicate features of human telomere biology as observed in various inflammatory disorders. It will now be critical to perform additional studies of telomere biology/telomerase activity in human leukocytes during natural aging and inflammation before this axis can be exploited for therapeutic benefit in the clinic.

## Conclusion

Immunosenescence is the culmination of a complex network of molecular processes. Despite intensive study over the last decade and improved understanding of the features of immunological aging, the molecular mediators of these events and the extent to which they interact remain poorly defined. Indeed, while the strong association of telomere length with cellular senescence has been known for decades, the direct/indirect relationship between telomerase activity and PRR signaling is only now coming to light. While the molecular basis of PRR interactions with telomerase activity has not yet been determined, better definition of this crosstalk will be essential to understanding the influence of PRRs and “inflammaging” on human hematopoiesis and tissue regeneration. The recently identified ability of stem cells to directly detect DAMPs and PAMPs *via* PRRs should lead to significant progress in developing methods of combating immunosenescence in a range of human pathologies. Together, these data underscore the importance of inflammaging as a major driver of senescence progression and reinforce the concept that an array of different pathways likely interact to determine the rate of this process (graphically represented in Figure 1). Recent analyses of complex data sets from large cohorts of elderly subjects and patients with various chronic disorders have already implicated key regulators of immunosenescence in determining clinical outcomes. However, a complete understanding of the molecular mechanisms at play will require more sophisticated animal models and validation in human studies before these can be effectively targeted for therapy in common diseases of aging and inflammation.

## Author Contributions

SSJ prepared the figure and wrote the manuscript, KB wrote the manuscript and prepared the table, TK and ZK advised clinical research interpretations, and JF conceptualized, wrote, and critically reviewed the manuscript.

## Conflict of Interest Statement

The authors declare that the research was conducted in the absence of any commercial or financial relationships that could be construed as a potential conflict of interest.

## References

[1] Lopez-Otin C Blasco MA Partridge L Serrano M Kroemer G. The hallmarks of aging. Cell (2013) 153(6):1194–217. 10.1016/j.cell.2013.05.039 23746838 PMC3836174

[2] Kirkwood TB. Understanding the odd science of aging. Cell (2005) 120(4):437–47. 10.1016/j.cell.2005.01.027 15734677

[3] Chiu CP Dragowska W Kim NW Vaziri H Yui J Thomas TE Differential expression of telomerase activity in hematopoietic progenitors from adult human bone marrow. Stem Cells (1996) 14(2):239–48. 10.1002/stem.140239 8991544

[4] Engelhardt M Kumar R Albanell J Pettengell R Han W Moore MA. Telomerase regulation, cell cycle, and telomere stability in primitive hematopoietic cells. Blood (1997) 90(1):182–93. 9207452

[5] Plunkett FJ Franzese O Finney HM Fletcher JM Belaramani LL Salmon M The loss of telomerase activity in highly differentiated CD8+CD28-CD27- T cells is associated with decreased Akt (Ser473) phosphorylation. J Immunol (2007) 178(12):7710–9. 10.4049/jimmunol.178.12.7710 17548608

[6] Akbar AN Vukmanovic-Stejic M. Telomerase in T lymphocytes: use it and lose it? J Immunol (2007) 178(11):6689–94. 10.4049/jimmunol.178.11.6689 17513711

[7] Palm W de Lange T. How shelterin protects mammalian telomeres. Annu Rev Genet (2008) 42:301–34. 10.1146/annurev.genet.41.110306.130350 18680434

[8] Agarwal S Busse PJ. Innate and adaptive immunosenescence. Ann Allergy Asthma Immunol (2010) 104(3):183–90. 10.1016/j.anai.2009.11.009 20377107

[9] Pulko V Davies JS Martinez C Lanteri MC Busch MP Diamond MS Human memory T cells with a naive phenotype accumulate with aging and respond to persistent viruses. Nat Immunol (2016) 17(8):966–75. 10.1038/ni.3483 27270402 PMC4955715

[10] Wikby A Nilsson BO Forsey R Thompson J Strindhall J Lofgren S The immune risk phenotype is associated with IL-6 in the terminal decline stage: findings from the Swedish NONA immune longitudinal study of very late life functioning. Mech Ageing Dev (2006) 127(8):695–704. 10.1016/j.mad.2006.04.003 16750842

[11] Wikby A Ferguson F Forsey R Thompson J Strindhall J Lofgren S An immune risk phenotype, cognitive impairment, and survival in very late life: impact of allostatic load in Swedish octogenarian and nonagenarian humans. J Gerontol A Biol Sci Med Sci (2005) 60(5):556–65. 10.1093/gerona/60.5.556 15972602

[12] Panda A Arjona A Sapey E Bai F Fikrig E Montgomery RR Human innate immunosenescence: causes and consequences for immunity in old age. Trends Immunol (2009) 30(7):325–33. 10.1016/j.it.2009.05.004 19541535 PMC4067971

[13] Fulop T Larbi A Douziech N Fortin C Guerard KP Lesur O Signal transduction and functional changes in neutrophils with aging. Aging Cell (2004) 3(4):217–26. 10.1111/j.1474-9728.2004.00110.x 15268755

[14] Morrisette-Thomas V Cohen AA Fulop T Riesco E Legault V Li Q Inflamm-aging does not simply reflect increases in pro-inflammatory markers. Mech Ageing Dev (2014) 139:49–57. 10.1016/j.mad.2014.06.005 25011077 PMC5881904

[15] De Martinis M Franceschi C Monti D Ginaldi L. Inflammation markers predicting frailty and mortality in the elderly. Exp Mol Pathol (2006) 80(3):219–27. 10.1016/j.yexmp.2005.11.004 16460728

[16] Chung SS Wu Y Okobi Q Adekoya D Atefi M Clarke O Proinflammatory cytokines IL-6 and TNF-α increased telomerase activity through NF-κB/STAT1/STAT3 activation, and withaferin A inhibited the signaling in colorectal cancer cells. Mediators Inflamm (2017) 2017:5958429 10.1155/2017/5958429 28676732 PMC5476880

[17] Zeng Y Nie C Min J Liu X Li M Chen H Novel loci and pathways significantly associated with longevity. Sci Rep (2016) 6:21243. 10.1038/srep21243 26912274 PMC4766491

[18] Bonafe M Olivieri F Cavallone L Giovagnetti S Mayegiani F Cardelli M A gender-dependent genetic predisposition to produce high levels of IL-6 is detrimental for longevity. Eur J Immunol (2001) 31(8):2357–61. 10.1002/1521-4141(200108)31:8<2357::AID-IMMU2357>3.0.CO;2-X 11500818

[19] Scheinecker C Smolen J Yasothan U Stoll J Kirkpatrick P Tocilizumab. Nat Rev Drug Discov (2009) 8(4):273–4. 10.1038/nrd2863 19337270

[20] Franceschi C Bonafe M Valensin S Olivieri F De Luca M Ottaviani E Inflamm-aging. An evolutionary perspective on immunosenescence.Ann N Y Acad Sci (2000) 908:244–54. 10.1111/j.1749-6632.2000.tb06651.x 10911963

[21] Franceschi C Capri M Monti D Giunta S Olivieri F Sevini F Inflammaging and anti-inflammaging: a systemic perspective on aging and longevity emerged from studies in humans. Mech Ageing Dev (2007) 128(1):92–105. 10.1016/j.mad.2006.11.016 17116321

[22] Tu W Rao S. Mechanisms underlying T cell immunosenescence: aging and cytomegalovirus infection. Front Microbiol (2016) 7:2111. 10.3389/fmicb.2016.02111 28082969 PMC5186782

[23] Peres A Bauer M da Cruz IB Nardi NB Chies JA. Immunophenotyping and T-cell proliferative capacity in a healthy aged population. Biogerontology (2003) 4(5):289–96. 10.1023/A:1026282917406 14618026

[24] Panda A Qian F Mohanty S van Duin D Newman FK Zhang L Age-associated decrease in TLR function in primary human dendritic cells predicts influenza vaccine response. J Immunol (2010) 184(5):2518–27. 10.4049/jimmunol.0901022 20100933 PMC3867271

[25] Fulop T Larbi A Witkowski JM Kotb R Hirokawa K Pawelec G Immunosenescence and cancer. Crit Rev Oncog (2013) 18(6):489–513. 10.1615/CritRevOncog.2013010597 24579731

[26] Solana R Tarazona R Gayoso I Lesur O Dupuis G Fulop T. Innate immunosenescence: effect of aging on cells and receptors of the innate immune system in humans. Semin Immunol (2012) 24(5):331–41. 10.1016/j.smim.2012.04.008 22560929

[27] Koch S Larbi A Ozcelik D Solana R Gouttefangeas C Attig S Cytomegalovirus infection: a driving force in human T cell immunosenescence. Ann N Y Acad Sci (2007) 1114:23–35. 10.1196/annals.1396.043 17986574

[28] Vasto S Colonna-Romano G Larbi A Wikby A Caruso C Pawelec G. Role of persistent CMV infection in configuring T cell immunity in the elderly. Immun Ageing (2007) 4:2. 10.1186/1742-4933-4-2 17376222 PMC1831794

[29] Jurk D Wilson C Passos JF Oakley F Correia-Melo C Greaves L Chronic inflammation induces telomere dysfunction and accelerates ageing in mice. Nat Commun (2014) 2:4172. 10.1038/ncomms5172 24960204 PMC4090717

[30] Sebastian C Herrero C Serra M Lloberas J Blasco MA Celada A. Telomere shortening and oxidative stress in aged macrophages results in impaired STAT5a phosphorylation. J Immunol (2009) 183(4):2356–64. 10.4049/jimmunol.0901131 19605693

[31] Zhu Y Armstrong JL Tchkonia T Kirkland JL. Cellular senescence and the senescent secretory phenotype in age-related chronic diseases. Curr Opin Clin Nutr Metab Care (2014) 17(4):324–8. 10.1097/MCO.0000000000000065 24848532

[32] von Zglinicki T Martin-Ruiz CM Telomeres as biomarkers for ageing and age-related diseases. Curr Mol Med (2005) 5(2):197–203. 10.2174/1566524053586545 15974873

[33] Franceschi C Garagnani P Vitale G Capri M Salvioli S Inflammaging and ‘Garb-aging’. Trends Endocrinol Metab (2017) 28(3):199–212. 10.1016/j.tem.2016.09.005 27789101

[34] Kordinas V Ioannidis A Chatzipanagiotou S The telomere/telomerase system in chronic inflammatory diseases. Cause or effect? Genes (Basel) (2016) 7(9):E60 10.3390/genes7090060 27598205 PMC5042391

[35] Lepperdinger G. Inflammation and mesenchymal stem cell aging. Curr Opin Immunol (2011) 23(4):518–24. 10.1016/j.coi.2011.05.007 21703839 PMC3167021

[36] Zhang Q Raoof M Chen Y Sumi Y Sursal T Junger W Circulating mitochondrial DAMPs cause inflammatory responses to injury. Nature (2010) 464(7285):104–7. 10.1038/nature08780 20203610 PMC2843437

[37] Pinti M Cevenini E Nasi M De Biasi S Salvioli S Monti D Circulating mitochondrial DNA increases with age and is a familiar trait: implications for “inflamm-aging”. Eur J Immunol (2014) 44(5):1552–62. 10.1002/eji.201343921 24470107

[38] Gupta GK Agrawal DK. CpG oligodeoxynucleotides as TLR9 agonists: therapeutic application in allergy and asthma. BioDrugs (2010) 24(4):225–35. 10.2165/11536140-000000000-00000 20623989

[39] Wang Z Lieberman PM. The crosstalk of telomere dysfunction and inflammation through cell-free TERRA containing exosomes. RNA Biol (2016) 13(8):690–5. 10.1080/15476286.2016.1203503 27351774 PMC4993293

[40] Lin L Park S Lakatta EG. RAGE signaling in inflammation and arterial aging. Front Biosci (Landmark Ed) (2009) 14:1403–13. 10.2741/3315 19273137 PMC2661616

[41] Samy RP Lim LH. DAMPs and influenza virus infection in ageing. Ageing Res Rev (2015) 24(Pt A):83–97. 10.1016/j.arr.2015.07.005 26200296

[42] Seneviratne AN Sivagurunathan B Monaco C. Toll-like receptors and macrophage activation in atherosclerosis. Clin Chim Acta (2012) 413(1–2):3–14. 10.1016/j.cca.2011.08.021 21884686

[43] Hovland A Jonasson L Garred P Yndestad A Aukrust P Lappegard KT The complement system and toll-like receptors as integrated players in the pathophysiology of atherosclerosis. Atherosclerosis (2015) 241(2):480–94. 10.1016/j.atherosclerosis.2015.05.038 26086357

[44] Falck-Hansen M Kassiteridi C Monaco C. Toll-like receptors in atherosclerosis. Int J Mol Sci (2013) 14(7):14008–23. 10.3390/ijms140714008 23880853 PMC3742229

[45] Meng X Ao L Song Y Babu A Yang X Wang M Expression of functional toll-like receptors 2 and 4 in human aortic valve interstitial cells: potential roles in aortic valve inflammation and stenosis. Am J Physiol Cell Physiol (2008) 294(1):C29–35. 10.1152/ajpcell.00137.2007 17942642

[46] Fyhrquist F Saijonmaa O Strandberg T. The roles of senescence and telomere shortening in cardiovascular disease. Nat Rev Cardiol (2013) 10(5):274–83. 10.1038/nrcardio.2013.30 23478256

[47] Gizard F Heywood EB Findeisen HM Zhao Y Jones KL Cudejko C Telomerase activation in atherosclerosis and induction of telomerase reverse transcriptase expression by inflammatory stimuli in macrophages. Arterioscler Thromb Vasc Biol (2011) 31(2):245–52. 10.1161/ATVBAHA.110.219808 21106948 PMC3025413

[48] Liu SC Wang SS Wu MZ Wu DC Yu FJ Chen WJ Activation of telomerase and expression of human telomerase reverse transcriptase in coronary atherosclerosis. Cardiovasc Pathol (2005) 14(5):232–40. 10.1016/j.carpath.2005.05.006 16168895

[49] Wagner KB Felix SB Riad A. Innate immune receptors in heart failure: side effect or potential therapeutic target? World J Cardiol (2014) 6(8):791–801. 10.4330/wjc.v6.i8.791 25228958 PMC4163708

[50] van der Harst P van der Steege G de Boer RA Voors AA Hall AS Mulder MJ Telomere length of circulating leukocytes is decreased in patients with chronic heart failure. J Am Coll Cardiol (2007) 49(13):1459–64. 10.1016/j.jacc.2007.01.027 17397675

[51] Bezemer GF Sagar S van Bergenhenegouwen J Georgiou NA Garssen J Kraneveld AD Dual role of toll-like receptors in asthma and chronic obstructive pulmonary disease. Pharmacol Rev (2012) 64(2):337–58. 10.1124/pr.111.004622 22407613

[52] Cordoba-Lanus E Cazorla-Rivero S Espinoza-Jimenez A de-Torres JP Pajares MJ Aguirre-Jaime A Telomere shortening and accelerated aging in COPD: findings from the BODE cohort. Respir Res (2017) 18(1):59. 10.1186/s12931-017-0547-4 28407775 PMC5390353

[53] Houben JM Mercken EM Ketelslegers HB Bast A Wouters EF Hageman GJ Telomere shortening in chronic obstructive pulmonary disease. Respir Med (2009) 103(2):230–6. 10.1016/j.rmed.2008.09.003 18945604

[54] Gabrilovich MI Walrath J van Lunteren J Nethery D Seifu M Kern JA Disordered toll-like receptor 2 responses in the pathogenesis of pulmonary sarcoidosis. Clin Exp Immunol (2013) 173(3):512–22. 10.1111/cei.12138 23668840 PMC3949639

[55] Guan JZ Maeda T Sugano M Oyama J Higuchi Y Suzuki T An analysis of telomere length in sarcoidosis. J Gerontol A Biol Sci Med Sci (2007) 62(11):1199–203. 10.1093/gerona/62.11.1199 18000138

[56] Maeda T Guan JZ Higuchi Y Oyama J Makino N. Aging-related alterations of subtelomeric methylation in sarcoidosis patients. J Gerontol A Biol Sci Med Sci (2009) 64(7):752–60. 10.1093/gerona/glp049 19414507

[57] Xiao X Zhao P Rodriguez-Pinto D Qi D Henegariu O Alexopoulou L Inflammatory regulation by TLR3 in acute hepatitis. J Immunol (2009) 183(6):3712–9. 10.4049/jimmunol.0901221 19710451 PMC3787866

[58] Broering R Montag M Jiang M Lu M Sowa JP Kleinehr K Corticosteroids shift the toll-like receptor response pattern of primary-isolated murine liver cells from an inflammatory to an anti-inflammatory state. Int Immunol (2011) 23(9):537–44. 10.1093/intimm/dxr048 21750146

[59] Kim S Park S Kim B Kwon J. Toll-like receptor 7 affects the pathogenesis of non-alcoholic fatty liver disease. Sci Rep (2016) 6:27849. 10.1038/srep27849 27279075 PMC4899790

[60] Mencin A Kluwe J Schwabe RF. Toll-like receptors as targets in chronic liver diseases. Gut (2009) 58(5):704–20. 10.1136/gut.2008.156307 19359436 PMC2791673

[61] Kitada T Seki S Kawakita N Kuroki T Monna T. Telomere shortening in chronic liver diseases. Biochem Biophys Res Commun (1995) 211(1):33–9. 10.1006/bbrc.1995.1774 7779103

[62] Mao TK Lian ZX Selmi C Ichiki Y Ashwood P Ansari AA Altered monocyte responses to defined TLR ligands in patients with primary biliary cirrhosis. Hepatology (2005) 42(4):802–8. 10.1002/hep.20859 16175622

[63] Sasaki M Ikeda H Yamaguchi J Nakada S Nakanuma Y. Telomere shortening in the damaged small bile ducts in primary biliary cirrhosis reflects ongoing cellular senescence. Hepatology (2008) 48(1):186–95. 10.1002/hep.22348 18536059

[64] Stanislawowski M Wierzbicki PM Golab A Adrych K Kartanowicz D Wypych J Decreased toll-like receptor-5 (TLR-5) expression in the mucosa of ulcerative colitis patients. J Physiol Pharmacol (2009) 60(Suppl 4):71–5. 20083854

[65] Franchimont D Vermeire S El Housni H Pierik M Van Steen K Gustot T Deficient host-bacteria interactions in inflammatory bowel disease? The toll-like receptor (TLR)-4 Asp299gly polymorphism is associated with Crohn’s disease and ulcerative colitis. Gut (2004) 53(7):987–92. 10.1136/gut.2003.030205 15194649 PMC1774122

[66] Friis-Ottessen M Bendix L Kolvraa S Norheim-Andersen S De Angelis PM Clausen OP. Telomere shortening correlates to dysplasia but not to DNA aneuploidy in longstanding ulcerative colitis. BMC Gastroenterol (2014) 14:8. 10.1186/1471-230X-14-8 24405569 PMC3893461

[67] Risques RA Lai LA Brentnall TA Li L Feng Z Gallaher J Ulcerative colitis is a disease of accelerated colon aging: evidence from telomere attrition and DNA damage. Gastroenterology (2008) 135(2):410–8. 10.1053/j.gastro.2008.04.008 18519043 PMC2574910

[68] O’Sullivan JN Bronner MP Brentnall TA Finley JC Shen WT Emerson S Chromosomal instability in ulcerative colitis is related to telomere shortening. Nat Genet (2002) 32(2):280–4. 10.1038/ng989 12355086

[69] Kinouchi Y Hiwatashi N Chida M Nagashima F Takagi S Maekawa H Telomere shortening in the colonic mucosa of patients with ulcerative colitis. J Gastroenterol (1998) 33(3):343–8. 10.1007/s005350050094 9658312

[70] Holzmann K Klump B Weis-Klemm M Hsieh CJ Borchard F Gregor M Telomerase activity in long-standing ulcerative colitis. Anticancer Res (2000) 20(5C):3951–5. 11268482

[71] Szebeni B Veres G Dezsofi A Rusai K Vannay A Bokodi G Increased mucosal expression of toll-like receptor (TLR)2 and TLR4 in coeliac disease. J Pediatr Gastroenterol Nutr (2007) 45(2):187–93. 10.1097/MPG.0b013e318064514a 17667714

[72] Kamycheva E Goto T Camargo CA Jr. Celiac disease autoimmunity is associated with leukocyte telomere shortening in older adults: the U.S. National Health and Nutrition Examination Survey. Exp Gerontol (2017) 89:64–8. 10.1016/j.exger.2017.01.003 28104447

[73] Rayavarapu S Coley W Kinder TB Nagaraju K. Idiopathic inflammatory myopathies: pathogenic mechanisms of muscle weakness. Skelet Muscle (2013) 3(1):13. 10.1186/2044-5040-3-13 23758833 PMC3681571

[74] Ponsot E Echaniz-Laguna A Delis AM Kadi F. Telomere length and regulatory proteins in human skeletal muscle with and without ongoing regenerative cycles. Exp Physiol (2012) 97(6):774–84. 10.1113/expphysiol.2011.063818 22366562

[75] Thwaites R Chamberlain G Sacre S. Emerging role of endosomal toll-like receptors in rheumatoid arthritis. Front Immunol (2014) 5:1. 10.3389/fimmu.2014.00001 24474949 PMC3893714

[76] Huang QQ Pope RM. The role of toll-like receptors in rheumatoid arthritis. Curr Rheumatol Rep (2009) 11(5):357–64. 10.1007/s11926-009-0051-z 19772831 PMC2913446

[77] Steer SE Williams FM Kato B Gardner JP Norman PJ Hall MA Reduced telomere length in rheumatoid arthritis is independent of disease activity and duration. Ann Rheum Dis (2007) 66(4):476–80. 10.1136/ard.2006.059188 17114192 PMC1856061

[78] Fujii H Shao L Colmegna I Goronzy JJ Weyand CM. Telomerase insufficiency in rheumatoid arthritis. Proc Natl Acad Sci U S A (2009) 106(11):4360–5. 10.1073/pnas.0811332106 19255426 PMC2657451

[79] Yudoh K Matsuno H Nezuka T Kimura T. Different mechanisms of synovial hyperplasia in rheumatoid arthritis and pigmented villonodular synovitis: the role of telomerase activity in synovial proliferation. Arthritis Rheum (1999) 42(4):669–77. 10.1002/1529-0131(199904)42:4<669::AID-ANR9>3.0.CO;2-V 10211880

[80] Tarhan F Vural F Kosova B Aksu K Cogulu O Keser G Telomerase activity in connective tissue diseases: elevated in rheumatoid arthritis, but markedly decreased in systemic sclerosis. Rheumatol Int (2008) 28(6):579–83. 10.1007/s00296-007-0472-9 17938929

[81] Kirchner M Sonnenschein A Schoofs S Schmidtke P Umlauf VN Mannhardt-Laakmann W. Surface expression and genotypes of toll-like receptors 2 and 4 in patients with juvenile idiopathic arthritis and systemic lupus erythematosus. Pediatr Rheumatol Online J (2013) 11(1):9. 10.1186/1546-0096-11-9 23497095 PMC3626865

[82] Prelog M Schwarzenbrunner N Sailer-Hock M Kern H Klein-Franke A Ausserlechner MJ Premature aging of the immune system in children with juvenile idiopathic arthritis. Arthritis Rheum (2008) 58(7):2153–62. 10.1002/art.23599 18576332

[83] Picarelli MM Danzmann LC Grun LK Junior NTR Lavandovsky P Guma F Arterial stiffness by oscillometric device and telomere length in juvenile idiopathic arthritis with no cardiovascular risk factors: a cross-sectional study. Pediatr Rheumatol Online J (2017) 15(1):34. 10.1186/s12969-017-0165-1 28472973 PMC5418721

[84] Ciechomska M Cant R Finnigan J van Laar JM O’Reilly S. Role of toll-like receptors in systemic sclerosis. Expert Rev Mol Med (2013) 15:e9. 10.1017/erm.2013.10 23985302

[85] Bhattacharyya S Varga J. Emerging roles of innate immune signaling and toll-like receptors in fibrosis and systemic sclerosis. Curr Rheumatol Rep (2015) 17(1):474. 10.1007/s11926-014-0474-z 25604573

[86] Yoshizaki A Iwata Y Komura K Ogawa F Hara T Muroi E CD19 regulates skin and lung fibrosis via toll-like receptor signaling in a model of bleomycin-induced scleroderma. Am J Pathol (2008) 172(6):1650–63. 10.2353/ajpath.2008.071049 18467694 PMC2408424

[87] Artlett CM Black CM Briggs DC Stevens CO Welsh KI. Telomere reduction in scleroderma patients: a possible cause for chromosomal instability. Br J Rheumatol (1996) 35(8):732–7. 10.1093/rheumatology/35.8.732 8761184

[88] Katayama Y Kohriyama K. Telomerase activity in peripheral blood mononuclear cells of systemic connective tissue diseases. J Rheumatol (2001) 28(2):288–91. 11246663

[89] Pan HF Wu GC Li WP Li XP Ye DQ. High mobility group box 1: a potential therapeutic target for systemic lupus erythematosus. Mol Biol Rep (2010) 37(3):1191–5. 10.1007/s11033-009-9485-7 19247800

[90] Celhar T Fairhurst AM. Toll-like receptors in systemic lupus erythematosus: potential for personalized treatment. Front Pharmacol (2014) 5:265. 10.3389/fphar.2014.00265 25538618 PMC4258990

[91] Rahman AH Eisenberg RA. The role of toll-like receptors in systemic lupus erythematosus. Springer Semin Immunopathol (2006) 28(2):131–43. 10.1007/s00281-006-0034-3 17047954

[92] Haque S Rakieh C Marriage F Ho P Gorodkin R Teh LS Shortened telomere length in patients with systemic lupus erythematosus. Arthritis Rheum (2013) 65(5):1319–23. 10.1002/art.37895 23400670

[93] Honda M Mengesha E Albano S Nichols WS Wallace DJ Metzger A Telomere shortening and decreased replicative potential, contrasted by continued proliferation of telomerase-positive CD8+CD28(lo) T cells in patients with systemic lupus erythematosus. Clin Immunol (2001) 99(2):211–21. 10.1006/clim.2001.5023 11318593

[94] O’Connor PM Lapointe TK Jackson S Beck PL Jones NL Buret AG. *Helicobacter pylori* activates calpain via toll-like receptor 2 to disrupt adherens junctions in human gastric epithelial cells. Infect Immun (2011) 79(10):3887–94. 10.1128/IAI.05109-11 21825064 PMC3187258

[95] Ishihara S Rumi MA Kadowaki Y Ortega-Cava CF Yuki T Yoshino N Essential role of MD-2 in TLR4-dependent signaling during *Helicobacter pylori*-associated gastritis. J Immunol (2004) 173(2):1406–16. 10.4049/jimmunol.173.2.1406 15240737

[96] Allison CC Kufer TA Kremmer E Kaparakis M Ferrero RL. *Helicobacter pylori* induces MAPK phosphorylation and AP-1 activation via a NOD1-dependent mechanism. J Immunol (2009) 183(12):8099–109. 10.4049/jimmunol.0900664 20007577

[97] Lee WP Hou MC Lan KH Li CP Chao Y Lin HC *Helicobacter pylori*-induced chronic inflammation causes telomere shortening of gastric mucosa by promoting PARP-1-mediated non-homologous end joining of DNA. Arch Biochem Biophys (2016) 606:90–8. 10.1016/j.abb.2016.07.014 27450718

[98] Kameshima H Yagihashi A Yajima T Watanabe N Ikeda Y *Helicobacter pylori* infection induces telomerase activity in premalignant lesions. Am J Gastroenterol (1999) 94(2):547–8. 10.1111/j.1572-0241.1999.00547.x 10022677

[99] Chen Z Cheng Y Xu Y Liao J Zhang X Hu Y Expression profiles and function of toll-like receptors 2 and 4 in peripheral blood mononuclear cells of chronic hepatitis B patients. Clin Immunol (2008) 128(3):400–8. 10.1016/j.clim.2008.04.006 18565796

[100] Isogawa M Robek MD Furuichi Y Chisari FV. Toll-like receptor signaling inhibits hepatitis B virus replication in vivo. J Virol (2005) 79(11):7269–72. 10.1128/JVI.79.11.7269-7272.2005 15890966 PMC1112123

[101] Tachtatzis PM Marshall A Aravinthan A Verma S Penrhyn-Lowe S Mela M Correction: chronic hepatitis B virus infection: the relation between hepatitis B antigen expression, telomere length, senescence, inflammation and fibrosis. PLoS One (2015) 10(7):e0134315 10.1371/journal.pone.0127511 26024529 PMC4449162

[102] Fan XG Huang Y Tang FQ Yi H. Telomerase activity of peripheral blood lymphocytes in patients with chronic hepatitis B. Immunol Lett (2000) 73(1):7–11. 10.1016/S0165-2478(00)00187-5 10963804

[103] Bailey KL Romberger DJ Katafiasz DM Heires AJ Sisson JH Wyatt TA TLR2 and TLR4 expression and inflammatory cytokines are altered in the airway epithelium of those with alcohol use disorders. Alcohol Clin Exp Res (2015) 39(9):1691–7. 10.1111/acer.12803 26208141 PMC4843766

[104] Uesugi T Froh M Arteel GE Bradford BU Thurman RG. Toll-like receptor 4 is involved in the mechanism of early alcohol-induced liver injury in mice. Hepatology (2001) 34(1):101–8. 10.1053/jhep.2001.25350 11431739

[105] Aida J Yokoyama A Izumiyama N Nakamura K Ishikawa N Poon SS Alcoholics show reduced telomere length in the oesophagus. J Pathol (2011) 223(3):410–6. 10.1002/path.2817 21171086

[106] Pace E Ferraro M Siena L Melis M Montalbano AM Johnson M Cigarette smoke increases toll-like receptor 4 and modifies lipopolysaccharide-mediated responses in airway epithelial cells. Immunology (2008) 124(3):401–11. 10.1111/j.1365-2567.2007.02788.x 18217953 PMC2440834

[107] Morla M Busquets X Pons J Sauleda J MacNee W Agusti AG. Telomere shortening in smokers with and without COPD. Eur Respir J (2006) 27(3):525–8. 10.1183/09031936.06.00087005 16507852

[108] Valdes AM Andrew T Gardner JP Kimura M Oelsner E Cherkas LF Obesity, cigarette smoking, and telomere length in women. Lancet (2005) 366(9486):662–4. 10.1016/S0140-6736(05)66630-5 16112303

[109] Yim HW Slebos RJ Randell SH Umbach DM Parsons AM Rivera MP Smoking is associated with increased telomerase activity in short-term cultures of human bronchial epithelial cells. Cancer Lett (2007) 246(1–2):24–33. 10.1016/j.canlet.2006.01.023 16517060

[110] Poulain-Godefroy O Le Bacquer O Plancq P Lecoeur C Pattou F Fruhbeck G Inflammatory role of toll-like receptors in human and murine adipose tissue. Mediators Inflamm (2010) 2010:823486. 10.1155/2010/823486 20339530 PMC2843862

[111] Creely SJ McTernan PG Kusminski CM Fisher FM Da Silva NF Khanolkar M Lipopolysaccharide activates an innate immune system response in human adipose tissue in obesity and type 2 diabetes. Am J Physiol Endocrinol Metab (2007) 292(3):E740–7. 10.1152/ajpendo.00302.2006 17090751

[112] Metcalf TU Cubas RA Ghneim K Cartwright MJ Grevenynghe JV Richner JM Global analyses revealed age-related alterations in innate immune responses after stimulation of pathogen recognition receptors. Aging Cell (2015) 14(3):421–32. 10.1111/acel.12320 25728020 PMC4406671

[113] Shaw AC Goldstein DR Montgomery RR. Age-dependent dysregulation of innate immunity. Nat Rev Immunol (2013) 13(12):875–87. 10.1038/nri3547 24157572 PMC4096436

[114] Nagai Y Garrett KP Ohta S Bahrun U Kouro T Akira S Toll-like receptors on hematopoietic progenitor cells stimulate innate immune system replenishment. Immunity (2006) 24(6):801–12. 10.1016/j.immuni.2006.04.008 16782035 PMC1626529

[115] Esplin BL Shimazu T Welner RS Garrett KP Nie L Zhang Q Chronic exposure to a TLR ligand injures hematopoietic stem cells. J Immunol (2011) 186(9):5367–75. 10.4049/jimmunol.1003438 21441445 PMC3086167

[116] Baldridge MT King KY Boles NC Weksberg DC Goodell MA. Quiescent haematopoietic stem cells are activated by IFN-gamma in response to chronic infection. Nature (2010) 465(7299):793–7. 10.1038/nature09135 20535209 PMC2935898

[117] Takizawa H Boettcher S Manz MG. Demand-adapted regulation of early hematopoiesis in infection and inflammation. Blood (2012) 119(13):2991–3002. 10.1182/blood-2011-12-380113 22246037

[118] Massberg S Schaerli P Knezevic-Maramica I Kollnberger M Tubo N Moseman EA Immunosurveillance by hematopoietic progenitor cells trafficking through blood, lymph, and peripheral tissues. Cell (2007) 131(5):994–1008. 10.1016/j.cell.2007.09.047 18045540 PMC2330270

[119] Griseri T McKenzie BS Schiering C Powrie F. Dysregulated hematopoietic stem and progenitor cell activity promotes interleukin-23-driven chronic intestinal inflammation. Immunity (2012) 37(6):1116–29. 10.1016/j.immuni.2012.08.025 23200826 PMC3664922

[120] De Luca K Frances-Duvert V Asensio MJ Ihsani R Debien E Taillardet M The TLR1/2 agonist PAM(3)CSK(4) instructs commitment of human hematopoietic stem cells to a myeloid cell fate. Leukemia (2009) 23(11):2063–74. 10.1038/leu.2009.155 19641520

[121] Zhao Y Ling F Wang HC Sun XH. Chronic TLR signaling impairs the long-term repopulating potential of hematopoietic stem cells of wild type but not Id1 deficient mice. PLoS One (2013) 8(2):e55552. 10.1371/journal.pone.0055552 23383338 PMC3562238

[122] Akala OO Clarke MF. Hematopoietic stem cell self-renewal. Curr Opin Genet Dev (2006) 16(5):496–501. 10.1016/j.gde.2006.08.011 16919448

[123] Orford K Scadden D. Deconstructing stem cell self-renewal: genetic insights into cell-cycle regulation. Nat Rev Genet (2008) 9(2):115–28. 10.1038/nrg2269 18202695

[124] Boiko JR Borghesi L. Hematopoiesis sculpted by pathogens: toll-like receptors and inflammatory mediators directly activate stem cells. Cytokine (2012) 57(1):1–8. 10.1016/j.cyto.2011.10.005 22079335 PMC3361504

[125] Serbina NV Hohl TM Cherny M Pamer EG. Selective expansion of the monocytic lineage directed by bacterial infection. J Immunol (2009) 183(3):1900–10. 10.4049/jimmunol.0900612 19596996 PMC2753883

[126] Takizawa H Regoes RR Boddupalli CS Bonhoeffer S Manz MG. Dynamic variation in cycling of hematopoietic stem cells in steady state and inflammation. J Exp Med (2011) 208(2):273–84. 10.1084/jem.20101643 21300914 PMC3039863

[127] Megias J Maneu V Salvador P Gozalbo D Gil ML. *Candida albicans* stimulates in vivo differentiation of haematopoietic stem and progenitor cells towards macrophages by a TLR2-dependent signalling. Cell Microbiol (2013) 15(7):1143–53. 10.1111/cmi.12104 23279268

[128] Schmid MA Takizawa H Baumjohann DR Saito Y Manz MG. Bone marrow dendritic cell progenitors sense pathogens via toll-like receptors and subsequently migrate to inflamed lymph nodes. Blood (2011) 118(18):4829–40. 10.1182/blood-2011-03-344960 21908421

[129] Ziegler P Boettcher S Takizawa H Manz MG Brummendorf TH. LPS-stimulated human bone marrow stroma cells support myeloid cell development and progenitor cell maintenance. Ann Hematol (2016) 95(2):173–8. 10.1007/s00277-015-2550-5 26555286

[130] Boettcher S Ziegler P Schmid MA Takizawa H van Rooijen N Kopf M Cutting edge: LPS-induced emergency myelopoiesis depends on TLR4-expressing nonhematopoietic cells. J Immunol (2012) 188(12):5824–8. 10.4049/jimmunol.1103253 22586037

[131] Bugl S Wirths S Radsak MP Schild H Stein P Andre MC Steady-state neutrophil homeostasis is dependent on TLR4/TRIF signaling. Blood (2013) 121(5):723–33. 10.1182/blood-2012-05-429589 23223360

[132] Colla S Ong DS Ogoti Y Marchesini M Mistry NA Clise-Dwyer K Telomere dysfunction drives aberrant hematopoietic differentiation and myelodysplastic syndrome. Cancer Cell (2015) 27(5):644–57. 10.1016/j.ccell.2015.04.007 25965571 PMC4596059

[133] Al-Ajmi N Saretzki G Miles C Spyridopoulos I. Dietary restriction ameliorates haematopoietic ageing independent of telomerase, whilst lack of telomerase and short telomeres exacerbates the ageing phenotype. Exp Gerontol (2014) 58:113–9. 10.1016/j.exger.2014.07.010 25038516

[134] Rossi DJ Bryder D Seita J Nussenzweig A Hoeijmakers J Weissman IL. Deficiencies in DNA damage repair limit the function of haematopoietic stem cells with age. Nature (2007) 447(7145):725–9. 10.1038/nature05862 17554309

[135] Chambers SM Shaw CA Gatza C Fisk CJ Donehower LA Goodell MA. Aging hematopoietic stem cells decline in function and exhibit epigenetic dysregulation. PLoS Biol (2007) 5(8):e201. 10.1371/journal.pbio.0050201 17676974 PMC1925137

[136] Bhattacharjee RN Banerjee B Akira S Hande MP. Telomere-mediated chromosomal instability triggers TLR4 induced inflammation and death in mice. PLoS One (2010) 5(7):e11873. 10.1371/journal.pone.0011873 20686699 PMC2912374

[137] Harberts E Gaspari AA. TLR signaling and DNA repair: are they associated? J Invest Dermatol (2013) 133(2):296–302. 10.1038/jid.2012.288 22931928 PMC8381358

[138] Liang Y Van Zant G Szilvassy SJ. Effects of aging on the homing and engraftment of murine hematopoietic stem and progenitor cells. Blood (2005) 106(4):1479–87. 10.1182/blood-2004-11-4282 15827136 PMC1895199

[139] Robbins CS Chudnovskiy A Rauch PJ Figueiredo JL Iwamoto Y Gorbatov R Extramedullary hematopoiesis generates Ly-6C(high) monocytes that infiltrate atherosclerotic lesions. Circulation (2012) 125(2):364–74. 10.1161/CIRCULATIONAHA.111.061986 22144566 PMC3263762

[140] Murakami S Yamamoto M Motohashi H. Hematopoietic stem and progenitor cell activation during chronic dermatitis provoked by constitutively active aryl-hydrocarbon receptor driven by keratin 14 promoter. Toxicol Sci (2014) 138(1):47–58. 10.1093/toxsci/kft273 24287212

[141] McGettrick AF O’Neill LA. Toll-like receptors: key activators of leucocytes and regulator of haematopoiesis. Br J Haematol (2007) 139(2):185–93. 10.1111/j.1365-2141.2007.06802.x 17897294

[142] Zhang H Rodriguez S Wang L Wang S Serezani H Kapur R Sepsis induces hematopoietic stem cell exhaustion and myelosuppression through distinct contributions of TRIF and MYD88. Stem Cell Reports (2016) 6(6):940–56. 10.1016/j.stemcr.2016.05.002 27264973 PMC4911503

[143] Shaw PJ Kan F Woo Ahn K Spellman SR Aljurf M Ayas M Outcomes of pediatric bone marrow transplantation for leukemia and myelodysplasia using matched sibling, mismatched related, or matched unrelated donors. Blood (2010) 116(19):4007–15. 10.1182/blood-2010-01-261958 20671124 PMC2981549

[144] Kauppila TE Kauppila JH Larsson NG. Mammalian mitochondria and aging: an update. Cell Metab (2017) 25(1):57–71. 10.1016/j.cmet.2016.09.017 28094012

[145] Li N Ragheb K Lawler G Sturgis J Rajwa B Melendez JA Mitochondrial complex I inhibitor rotenone induces apoptosis through enhancing mitochondrial reactive oxygen species production. J Biol Chem (2003) 278(10):8516–25. 10.1074/jbc.M210432200 12496265

[146] Shimada K Crother TR Karlin J Dagvadorj J Chiba N Chen S Oxidized mitochondrial DNA activates the NLRP3 inflammasome during apoptosis. Immunity (2012) 36(3):401–14. 10.1016/j.immuni.2012.01.009 22342844 PMC3312986

[147] Passos JF Saretzki G Ahmed S Nelson G Richter T Peters H Mitochondrial dysfunction accounts for the stochastic heterogeneity in telomere-dependent senescence. PLoS Biol (2007) 5(5):e110. 10.1371/journal.pbio.0050110 17472436 PMC1858712

[148] Handa H Matsushima T Nishimoto N Inoue M Saitoh T Yokohama A Flow cytometric detection of human telomerase reverse transcriptase (hTERT) expression in a subpopulation of bone marrow cells. Leuk Res (2010) 34(2):177–83. 10.1016/j.leukres.2009.06.010 19604579

[149] Narducci ML Grasselli A Biasucci LM Farsetti A Mule A Liuzzo G High telomerase activity in neutrophils from unstable coronary plaques. J Am Coll Cardiol (2007) 50(25):2369–74. 10.1016/j.jacc.2007.08.048 18154960

[150] Lepreux S Doudnikoff E Aubert I Bioulac-Sage P Bloch B Martin-Negrier ML. Cytoplasmic expression of human telomerase catalytic protein (hTERT) in neutrophils: an immunoelectron microscopy study. Ultrastruct Pathol (2008) 32(5):178–83. 10.1080/01913120802034504 18958789

[151] Kim YW Byzova TV Oxidative stress in angiogenesis and vascular disease. Blood (2014) 123(5):625–31. 10.1182/blood-2013-09-512749 24300855 PMC3907751

[152] Xie Z Jay KA Smith DL Zhang Y Liu Z Zheng J Early telomerase inactivation accelerates aging independently of telomere length. Cell (2015) 160(5):928–39. 10.1016/j.cell.2015.02.002 25723167 PMC4496004

[153] da Silva MS Segatto M Pavani RS Gutierrez-Rodrigues F Bispo VD de Medeiros MH Consequences of acute oxidative stress in *Leishmania amazonensis*: from telomere shortening to the selection of the fittest parasites. Biochim Biophys Acta (2017) 1864(1):138–50. 10.1016/j.bbamcr.2016.11.001 27836509

[154] Harley CB Futcher AB Greider CW. Telomeres shorten during ageing of human fibroblasts. Nature (1990) 345(6274):458–60. 10.1038/345458a0 2342578

[155] Indran IR Hande MP Pervaiz S. hTERT overexpression alleviates intracellular ROS production, improves mitochondrial function, and inhibits ROS-mediated apoptosis in cancer cells. Cancer Res (2011) 71(1):266–76. 10.1158/0008-5472.CAN-10-1588 21071633

[156] Nitta E Yamashita M Hosokawa K Xian M Takubo K Arai F Telomerase reverse transcriptase protects ATM-deficient hematopoietic stem cells from ROS-induced apoptosis through a telomere-independent mechanism. Blood (2011) 117(16):4169–80. 10.1182/blood-2010-08-297390 21297001

[157] Gursel I Gursel M Yamada H Ishii KJ Takeshita F Klinman DM. Repetitive elements in mammalian telomeres suppress bacterial DNA-induced immune activation. J Immunol (2003) 171(3):1393–400. 10.4049/jimmunol.171.3.1393 12874230

[158] Yin L Hubbard AK Giardina C. NF-kappa B regulates transcription of the mouse telomerase catalytic subunit. J Biol Chem (2000) 275(47):36671–5. 10.1074/jbc.M007378200 10970902

[159] Xu D Erickson S Szeps M Gruber A Sangfelt O Einhorn S Interferon alpha down-regulates telomerase reverse transcriptase and telomerase activity in human malignant and nonmalignant hematopoietic cells. Blood (2000) 96(13):4313–8. 11110707

[160] Ghosh A Saginc G Leow SC Khattar E Shin EM Yan TD Telomerase directly regulates NF-kappaB-dependent transcription. Nat Cell Biol (2012) 14(12):1270–81. 10.1038/ncb2621 23159929

[161] Hoffmeyer K Raggioli A Rudloff S Anton R Hierholzer A Del Valle I Wnt/beta-catenin signaling regulates telomerase in stem cells and cancer cells. Science (2012) 336(6088):1549–54. 10.1126/science.1218370 22723415

[162] Wu XQ Yang Y Li WX Cheng YH Li XF Huang C Telomerase reverse transcriptase acts in a feedback loop with NF-kappaB pathway to regulate macrophage polarization in alcoholic liver disease. Sci Rep (2016) 6:18685 10.1038/srep18685 26725521 PMC4698632

[163] Rentoukas E Tsarouhas K Kaplanis I Korou E Nikolaou M Marathonitis G Connection between telomerase activity in PBMC and markers of inflammation and endothelial dysfunction in patients with metabolic syndrome. PLoS One (2012) 7(4):e35739. 10.1371/journal.pone.0035739 22558213 PMC3338458

[164] Deelen J Beekman M Codd V Trompet S Broer L Hagg S Leukocyte telomere length associates with prospective mortality independent of immune-related parameters and known genetic markers. Int J Epidemiol (2014) 43(3):878–86. 10.1093/ije/dyt267 24425829 PMC4052133

[165] Calado RT Dumitriu B. Telomere dynamics in mice and humans. Semin Hematol (2013) 50(2):165–74. 10.1053/j.seminhematol.2013.03.030 23956466 PMC3742037

